# Clinical capabilities of graduates of an outcomes-based integrated medical program

**DOI:** 10.1186/1472-6920-12-23

**Published:** 2012-06-11

**Authors:** Helen A Scicluna, Michael C Grimm, Anthony J O’Sullivan, Peter Harris, Louis S Pilotto, Philip D Jones, H Patrick McNeil

**Affiliations:** 1 Faculty of Medicine, University of New South Wales, Sydney, Australia; 2 St George Clinical School, Faculty of Medicine, University of New South Wales, Sydney, Australia; 3 Rural Clinical School, Faculty of Medicine, University of New South Wales, Sydney, Australia; 4 South Western Sydney Clinical School, Faculty of Medicine, University of New South Wales, Sydney, Australia

## Abstract

**Background:**

The University of New South Wales (UNSW) Faculty of Medicine replaced its old content-based curriculum with an innovative new 6-year undergraduate entry outcomes-based integrated program in 2004. This paper is an initial evaluation of the perceived and assessed clinical capabilities of recent graduates of the new outcomes-based integrated medical program compared to benchmarks from traditional content-based or process-based programs.

**Method:**

Self-perceived capability in a range of clinical tasks and assessment of medical education as preparation for hospital practice were evaluated in recent graduates after 3 months working as junior doctors. Responses of the 2009 graduates of the UNSW’s new outcomes-based integrated medical education program were compared to those of the 2007 graduates of UNSW’s previous content-based program, to published data from other Australian medical schools, and to hospital-based supervisor evaluations of their clinical competence.

**Results:**

Three months into internship, graduates from UNSW’s new outcomes-based integrated program rated themselves to have good clinical and procedural skills, with ratings that indicated significantly greater capability than graduates of the previous UNSW content-based program. New program graduates rated themselves significantly more prepared for hospital practice in the confidence (reflective practice), prevention (social aspects of health), interpersonal skills (communication), and collaboration (teamwork) subscales than old program students, and significantly better or equivalent to published benchmarks of graduates from other Australian medical schools. Clinical supervisors rated new program graduates highly capable for teamwork, reflective practice and communication.

**Conclusions:**

Medical students from an outcomes-based integrated program graduate with excellent self-rated and supervisor-evaluated capabilities in a range of clinically-relevant outcomes. The program-wide curriculum reform at UNSW has had a major impact in developing capabilities in new graduates that are important for 21^st^ century medical practice.

## Background

The world-wide reform of medical education over the past 40 years that saw widespread adoption of curricula designed around an educational *process*, for example problem-based learning, has been highly controversial, with ongoing discussion about the educational outcomes of graduates of these curricula compared to previous content-based curricula [1-5]. Yet the influences that drove medical education reform away from content-based curricula remain – educational research showing the benefits of integrated and within context learning, ever-increasing medical knowledge, the information technology revolution, and demands to ensure learning in patient safety and professionalism. These influences prevent a return to content-based curricula that typically also include assessments of only a limited range of educational outcomes.

Some medical schools have responded by adopting an *outcomes*-based approach to curriculum design [6-9], in which a wider scope of desired competencies or capabilities of graduates represents the starting point for design of the program and its assessment system. Moreover, peak regulatory bodies such as the Graduate Medical Council [10], the Royal College of Physicians and Surgeons of Canada [11], and the Australian Medical Council [12], have prepared outcomes-based standards for medical school accreditation. Graduates of these programs are now emerging, and the challenge for schools is to evaluate whether their graduates are indeed achieving the key educational outcomes they espouse, and whether these outcomes translate into better clinical and professional capabilities for 21^st^ century practitioners.

It should be recognised at the outset that evaluating a holistic concept such as clinical capability is challenging, and inferring an effect from the undergraduate experience in the short and long term even more so [13]. Differences in junior doctors graduating from various medical curricula tend to diminish over time, emphasising the importance of experiential learning that occurs in all practitioners in the work place after graduation. Thus, an important time to evaluate the value of university education is soon after graduation. Self-perceptions by recent graduates of clinical capability and how their school prepared them for hospital practice are well-validated outcomes, and accepted as surrogates of more independent, yet harder to obtain evaluations [14-17]. Moreover, perceived self-efficacy may translate directly to improved performance in the stressful circumstances of the newly graduated doctors’ workplace, as those with high levels of perceived self-efficacy have been shown to be more likely to cope with the demands of jobs and tasks in the work environment [18].

In 2004, the University of New South Wales (UNSW) Faculty of Medicine replaced its old content-based curriculum with an innovative new 6-year undergraduate entry outcomes-based program, structured around the explicit development in students of eight educational outcomes that include five relatively generic capabilities (effective communication, teamwork, self direction, ethical practice, and reflective practice) as well as three traditional discipline-specific capabilities (scientific basis of medicine, social aspects of health, and patient assessment and management) [8]. The reader is referred to other sources that outline the new UNSW program in more detail, how it differs from other integrated medical programs [8], how learning activities foster development of generic capabilities [17], and how its novel assessment system drives learning in, and measures achievement of all eight educational outcomes [19]. In this paper, we report an initial evaluation of self-perceived and supervisor-evaluated clinical capabilities of the new UNSW program’s first graduating cohort, and compare results to a previous cohort and to historical benchmarks.

## Methods

We constructed a 66-item Clinical Capability Questionnaire (CCQ) in two parts (Appendix 1). Part 1 comprised 46 items listing a range of clinically-relevant tasks with 5 possible responses to the question ‘Please indicate at which level you believe you can perform the following skills on a patient at the present time’, that ranged from ‘I did not try the skill during Medical School or Internship’ = 1, ‘I tried the skill but I cannot perform it’ = 2, ‘I tried the skill and I can perform it supervised’ = 3, ‘I tried the skill and I can perform it unsupervised’ = 4, to ‘I tried the skill and I mastered it’ = 5. Responses of 4 or 5 were considered as evidence of good capability. The 46 items were divided into 4 subscales of items assessing clinical skills (18 items), procedural skills (14 items), operational management skills (9 items), and administrative tasks (5 items). These items evaluate outcomes in the ‘patient assessment and management’ capability of the UNSW medical program. Cronbach’s alpha co-efficients for each subscale were from 0.82 to 0.85, indicating good internal consistency on the items in the subscale.

Part 2 comprised 4 subscales selected from the 8 subscales of the Preparation for Hospital Practice Questionnaire (PHPQ), an instrument that has been previously used by medical schools to assess their graduates’ clinical capabilities [16]. The PHPQ subscales of interpersonal skills, confidence, collaboration and prevention were selected for inclusion in this study as each of these subscales correspond respectively to outcomes in the ‘communication’, ‘reflective practice’, ‘teamwork’, and ‘social aspects of health’ capabilities of the UNSW assessment system. Participants were requested to respond to the question ‘Please indicate the level at which you believe that medical school prepared you to’ with a 6-point scale ranging from ‘very inadequately’ = 1, ‘inadequately’ =2, ‘somewhat inadequately’ = 3, ‘somewhat adequately’ = 4, ‘adequately’ = 5, or ‘very adequately’ =6.

UNSW medical students who graduated in 2007 from the old content-based program (referred to as the old medical program), and those who graduated in 2009 from the new outcomes-based integrated program (referred to as the new medical program), were contacted in March (2008 and 2010 respectively), approximately 3 months after their graduation and invited to complete the on-line CCQ questionnaire (UNSW Ethics approval 2007/9/746). Graduates commence working as junior doctors in January. Valid responses were received from 92 of the 2007 cohort and 55 of the 2009 cohort, representing response rates of 43% and 27% respectively. Being the first cohort of a new curriculum, the 2009 cohort had been involved in a number of questionnaires earlier in their program, which probably accounts for the lower response rate in this post-graduation evaluation. Nevertheless, demographics of the respondents showed no significant differences from their respective cohorts suggesting responses were representative. Respondents had a mean age of 24.4 and 23.8 years for 2007 and 2009 respectively, and 58.7% and 56.4% were female, whereas the whole 2007 cohort had a mean age of 24.4 years and 54.4% female and the 2009 cohort had a mean age of 24.4 years and 58.9% female. Responses to Part 2 of the CCQ were compared to published data from the 2002 (n = 37) and 2004 (n = 35) graduating cohorts from the University of Tasmania Medical School [20], and the 1994 graduating cohorts from the University of Newcastle (n = 52), and the Universities of Sydney and NSW (n = 87 combined) [16].

To compare the 2009 graduating cohort’s self-reported data with external assessments of clinical capability made by their hospital–based supervisors, we obtained their consent prior to graduation in November 2009 (UNSW Ethics approval 2009/759) to retrieve their self-ratings and the ratings made by hospital-based senior clinician supervisors using the NSW Prevocational Progress Review Form End of Term Summative Assessment (PRF-ETSA) [21]. The PRF-ETSA requires supervisors and junior doctors to rate the junior doctor’s performance on 18 questions relating to clinical management, communication and professionalism on a 4 point scale ranging from clearly below the expected level = 1, borderline/requires assistance = 2, at expected level = 3, and clearly above the expected level = 4. A final question rates the junior doctor’s overall performance. The 18 questions were assigned into seven subscales that align with specific UNSW graduate capabilities (Appendix 2). Completed PRF-ETSA forms were received that provided self and supervisor evaluations of 109 junior doctors who graduated from the new UNSW medicine program; these 109 included the 55 respondents to the CCQ survey.

We analysed the data using Predictive Analytics Software (PASW - version 18). Mean scores for the subscales of both parts of the CCQ were calculated by averaging the raw scores for individual items. In analysing the PRF-ETSA data, the percentage of junior doctors who were clearly above the expected level (a score of 4) was calculated for the individual items and then averaged to calculate the percentage for the subscale. Independent t-tests were used to investigate the differences between 2009 and 2007 UNSW graduates on the CCQ and between supervisors’ ratings and junior doctors’ self-ratings on the PRF-ETSA. A one sample t-test was used to compare the 2009 UNSW graduates’ mean score on subscales of the PHPQ against the means for the other graduating cohorts. P values of <0.05 were considered significant.

## Results

**Figure 1:**
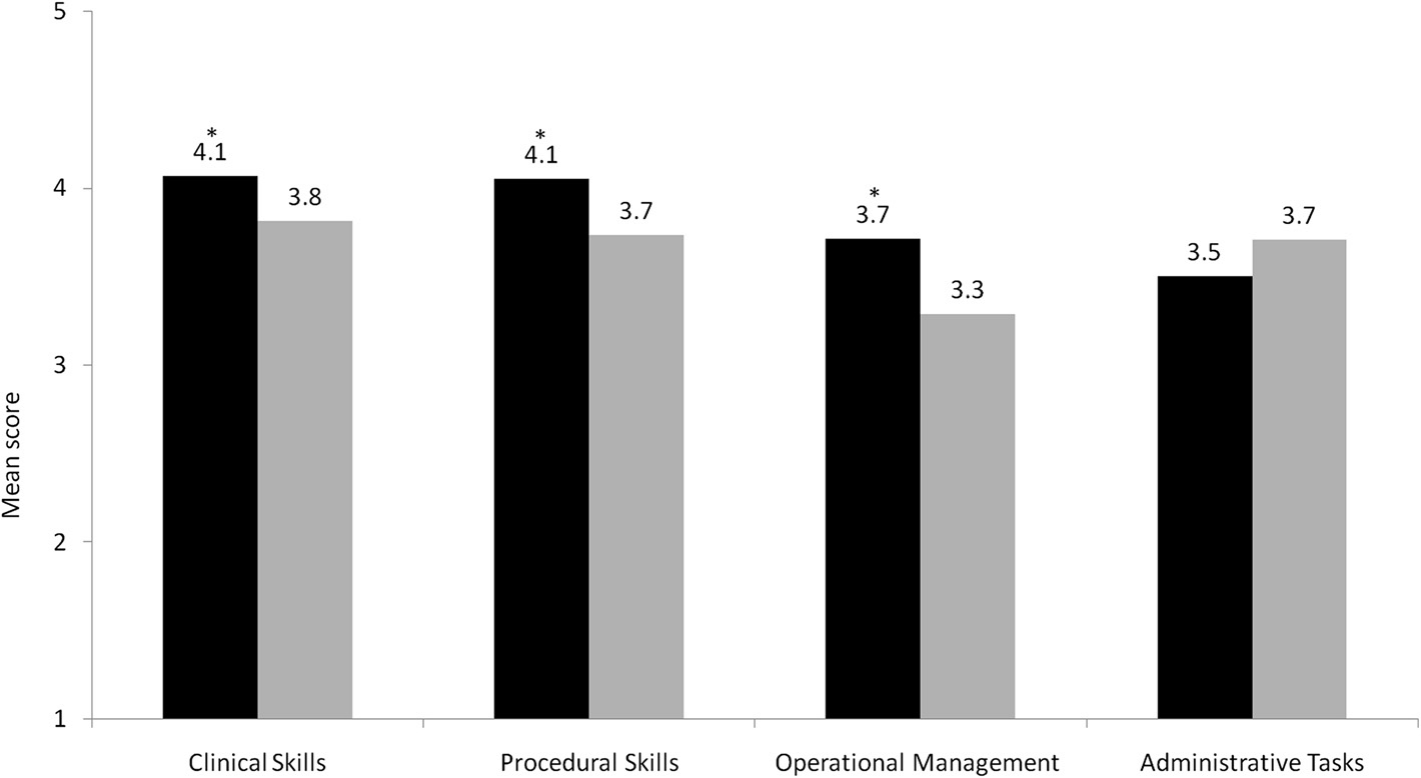
**Mean self-perceived capability on 46 clinical tasks of 2009 UNSW outcomes-based curriculum graduates (black bars) and 2007 UNSW content-based curriculum graduates (grey bars) when evaluated 3 months into internship.** * significant difference.

The mean scores of new program 2009 UNSW graduates for the clinical and procedural skills subscales were 4.1 (SD = 0.3) and 4.1 (SD = 0.4) respectively (Figure 1), indicating good self-perceived capability, given that a score of 4 equates to being able to perform the skill unsupervised. Mean scores of the new UNSW program 2009 cohort for the operational management skills and administrative tasks were lower at 3.7 (SD = 0.5) and 3.5 (SD = 0.7) respectively. Compared to responses of old program (2007) graduates, new program (2009) graduates rated themselves significantly more capable for the clinical (*P* < 0.001), procedural (*P* = 0.002), and operational management skills (*P* < 0.001) subscales, whereas there was no difference for the administrative tasks subscale (*P* = 0.126) (Figure 1).

**Figure 2:**
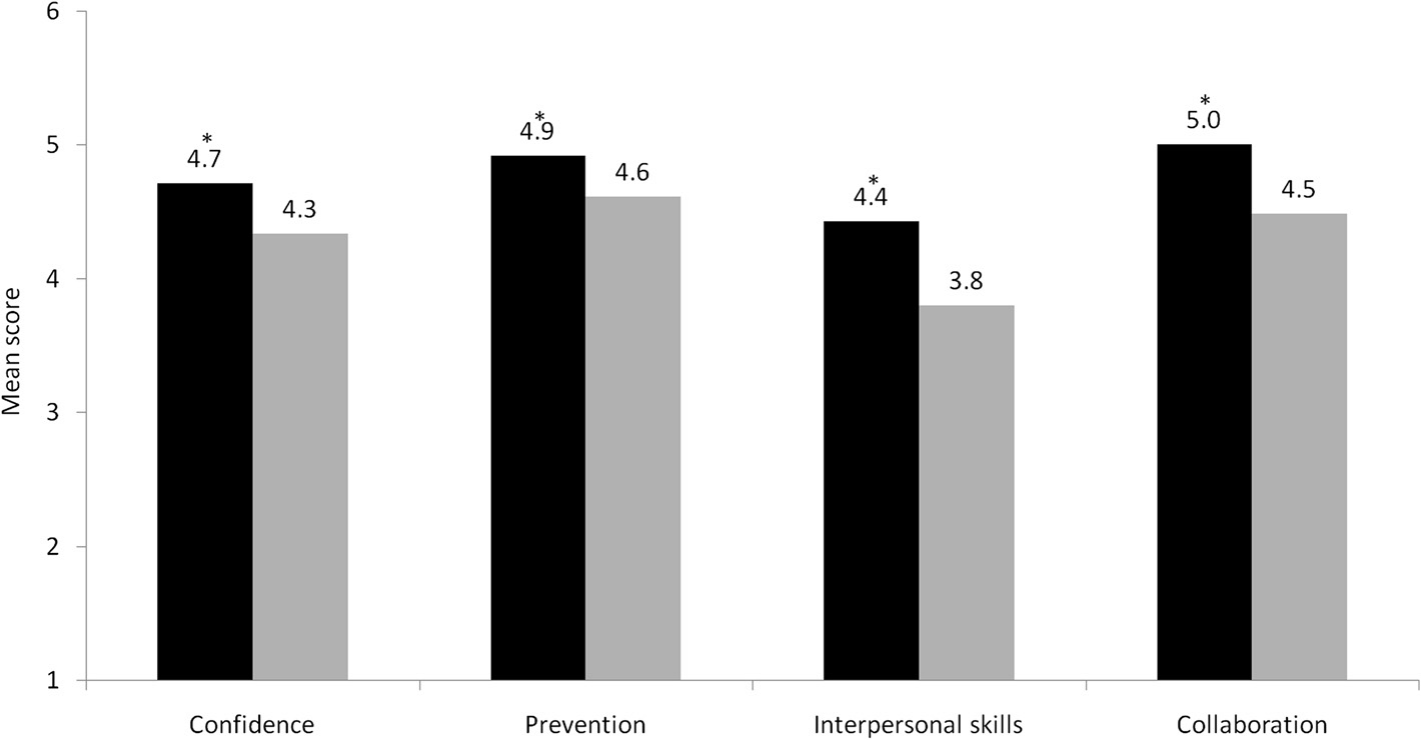
**Mean self-reported preparedness for hospital practice of 2009 UNSW outcomes-based curriculum graduates (black bars) and 2007 UNSW content-based curriculum graduates (grey bars) when evaluated 3 months into internship.** * significant difference.

**Figure 3:**
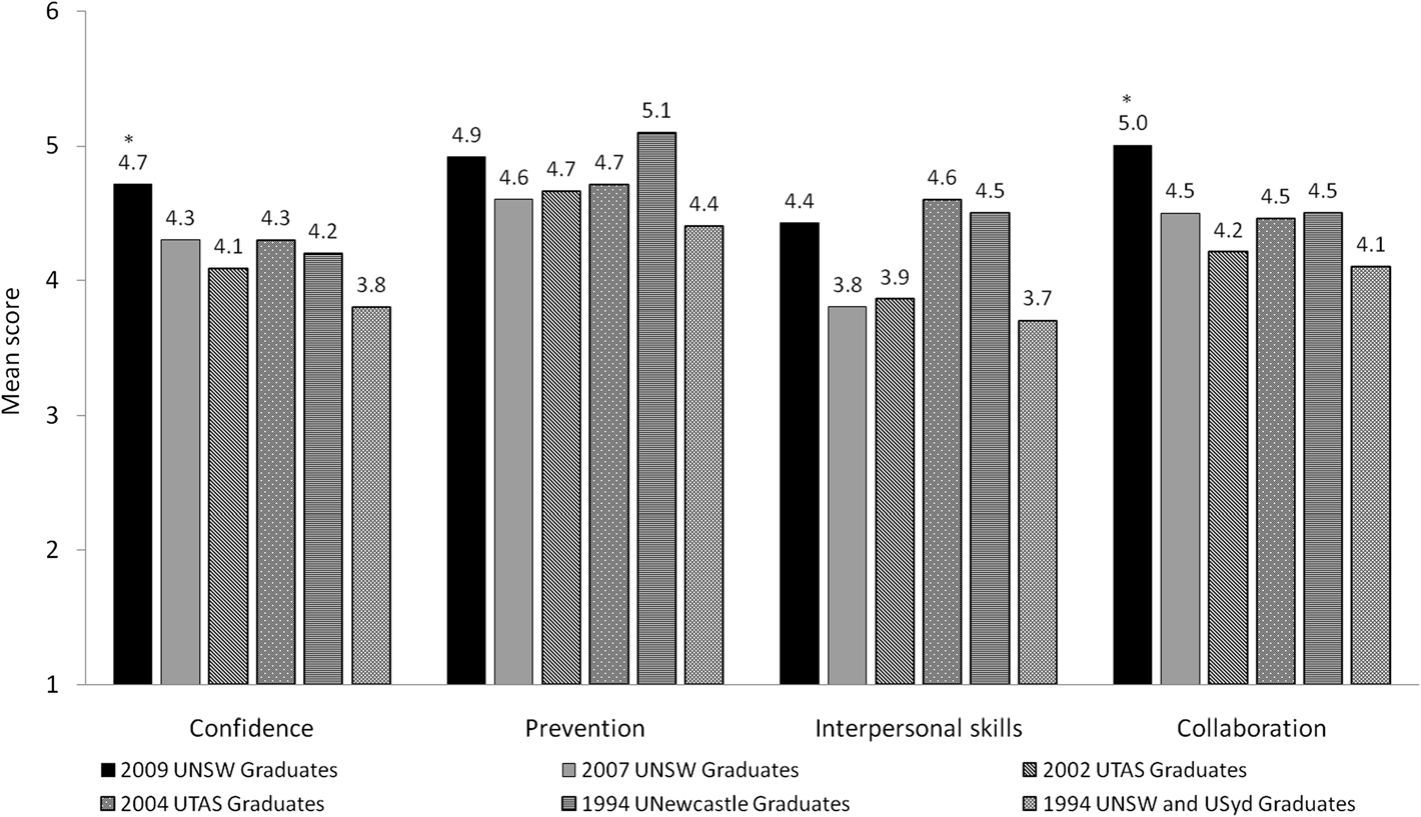
**Mean self-reported preparedness for hospital practice of 2009 and 2007 UNSW graduates evaluated 3 months into internship, compared to historical benchmarks from 2002 and 2004 graduates of the University of Tasmania (assessed ~3 months into internship) and 1994 graduates of the Universities of Newcastle, UNSW and Sydney (assessed at 6 months into internship).** * significantly higher scores for 2009 UNSW graduates compared to the highest score of other cohorts.

Graduates of the new UNSW program rated themselves better prepared for hospital practice on all 4 subscales of the PHPQ compared to the 2007 graduating cohort (Figure 2), (*P* < 0.001 for interpersonal and collaboration and *P* = 0.003 for confidence and prevention). The areas where new program graduates improved most substantially were in the ‘inter-personal skills’ subscale, which evaluates advanced clinical communication skills, and the ‘collaboration’ subscale, which measures inter-professional health teamwork (Appendix 1). When compared to published data of responses on the PHPQ from other universities, graduates of the new outcomes-based UNSW program had equivalent ratings on the ‘prevention’ and ‘interpersonal skills’ subscales, but significantly higher ratings (*P* < 0.001) than published benchmarks for the ‘confidence’ and ‘collaboration’ subscales (Figure 3). Items in the ‘confidence’ subscale evaluate learning in the ‘reflective practice’ capability as defined in UNSW’s assessment system.

**Figure 4:**
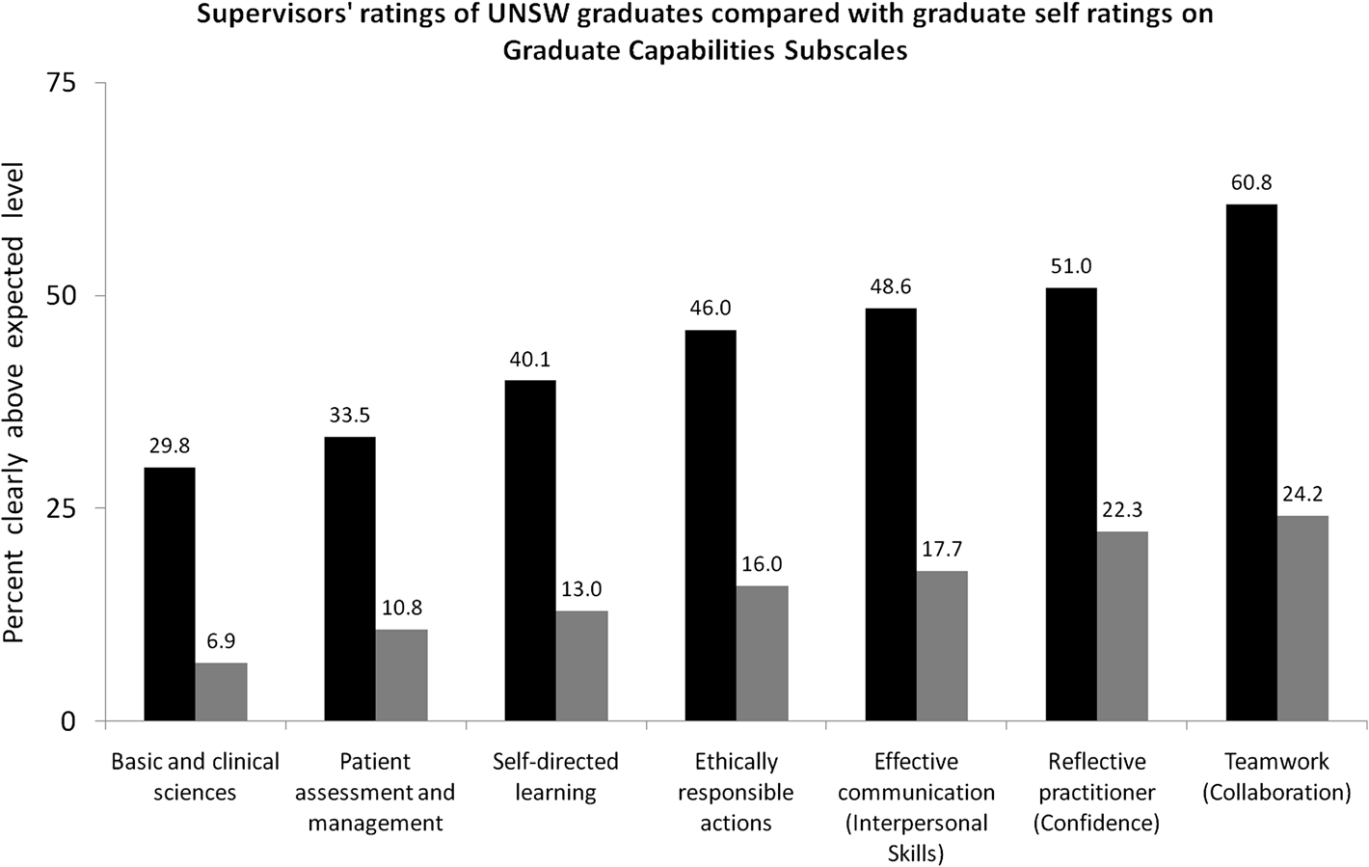
**Mean percentage of 2009 UNSW graduates evaluated as ‘clearly above expected level’ on the PRF-ETSA by hospital-based supervisors (black bars) compared to the graduate’s self ratings (grey bars).** Differences between supervisor and graduate self-ratings were significantly different for all capabilities.

Hospital-based supervisors rated new program graduates highest for the teamwork, reflective practice, and effective communication capabilities with 60.8%, 51% and 48.6% of graduates rated ‘clearly above expected level’ respectively (Figure 4). The high ranking of new program graduates in these three capabilities by supervisors, aligned closely with the capabilities that new program graduates expressed high self-perception in the PHPQ (Appendix 2). Moreover, supervisors consistently rated their junior doctors more capable than the junior doctors’ self-ratings (Figure 4), indicating that self-perceptions do not represent over-estimations of clinical competence.

## Discussion

Outcomes-based curricula represent a new approach to medical education [22]. Although similar to other contemporary integrated programs that use clinical contexts for learning and employ small group learning processes, outcomes-based programs are fundamentally different in their primary emphasis on achievement of defined learning outcomes, rather than adherence to a particular learning process. Evaluations of these new programs using a range of methodologies are important to determine effectiveness in the short and long term.

In this paper, we present an initial assessment of the clinical capabilities of the first cohort of UNSW’s outcomes-based program using graduate self-perceptions and external evaluations of clinical capability as measures of learning outcomes. The limitations of using self-perceived ratings as an outcome measure are acknowledged. However, perceived self-efficacy is critical to the success of action based on acquired knowledge and skills: the stronger that perception, the more ambitious are the goals individuals set for themselves and the more committed their approach to them [23], supporting the notion that perceived self-efficacy translates to real performance. There is also a comprehensive body of research showing that properly gathered student perceptions data concerning the effectiveness of many aspects of their education have validity for drawing evaluative conclusions about educational quality [14,17], a finding supported by more circumscribed studies which show good positive correlations between student perceptions and other assessments of teaching effectiveness and/or academic outcomes [15,24]. The self-perception data in this study are validated by the external evaluations made by experienced hospital-based supervisors, and supports previous findings that students consistently rate themselves lower than their supervisors [24]. Moreover, the validity of our results is strengthened since we used identical methodology to compare two very similar cohorts, with no significant differences in age, gender or maturity.

Overall, we consider the results to be a provisional endorsement of the new outcomes-based curriculum at UNSW with evidence that graduates report significantly better self-perceived capability across a range of clinical tasks most relevant to the ‘patient assessment and management’ capability, than graduates of the old content-based curriculum who completed their study two years earlier. Furthermore, new program UNSW graduates felt significantly better prepared for hospital practice than old program graduates on all 4 subscales evaluated, and on 2 of the 4 subscales evaluated when compared to graduates from other medical schools who completed the PHPQ at a similar stage. These findings provide evidence that graduates from an outcomes-based integrated program have well-developed capability in social aspects of health (prevention subscale), and clinical communication (interpersonal skills subscale), and superior capability in teamwork (collaboration subscale) and reflective practice (confidence subscale). The significant improvements in these latter two areas provide support for the view that capabilities such as ‘reflective practice’, and ‘teamwork’, can indeed be learned if explicitly incorporated into teaching and learning activities, and in particular if they are appropriately assessed [19,25].

The reasons for improved self-perceptions and positive external evaluations of clinical capability in the new UNSW program are multi-factorial relating to curriculum structure, teaching methodology and use of information technology, as well as a stronger emphasis on the teaching and learning of capabilities such as reflective practice and self-directed learning [8,17]. New program students have early clinical exposure with more emphasis on case-based teaching [26], and the use of innovative assessment tools such as the mini-CEX [27], as strategies which further facilitate clinical learning [28]. Communication skills and motivational interviewing are taught in tandem with clinical skills in the new UNSW program, a strategy which has been shown to positively enhance clinical performance [29]. Teamwork, group projects, peer-feedback and an emphasis on team dynamics are all integral to the learning and assessment process, so it was expected that this cohort’s collaborative skills would be stronger [25].

## Conclusions

The shift from a discipline or content-based curriculum to an outcomes-based program has resulted in significantly higher perceptions of clinical competence in our first graduating cohort, particularly in the more generic capability areas of teamwork, reflective practice, and clinical communication. These higher self-perceptions of clinical competence have been provisionally validated by hospital-based supervisors’ evaluations of the same cohort’s capabilities in the work place.

## Competing interests

The authors declare that they have no competing interests.

## Authors' contributions

HAS collected data, undertook statistical analysis and with HPM analysed the data and drafted the manuscript. HAS, MCG, LSP and PDJ developed the Clinical Capability Questionnaire and edited the manuscript. AJO and PH provided intellectual input into the study and helped draft the manuscript. All authors read and approved the final manuscript.

## Appendix 1: Clinical capability questionnaire

**Part 1:** Please indicate at which level you believe you can perform the following skills on a patient at the present time:

1. Recognition of a sick patient

2. Sterile dressing

3. Eversion of upper eye lid

4. Mouth examination

5. Knee examination

6. Ankle examination

7. Neurological examination

8. Blood pressure measurement

9. Pulse measurement

10. Blood glucose examination

11. IV fluid orders

12. Heart auscultation

13. Lung auscultation

14. Spirometry

15. Administration of a nebulised medication

16. Lymph node palpation

17. Abdominal palpation

18. Breast examination

19. Gynaecological examination

20. PAP smear

21. Cervical swab

22. Urine dipstick test

23. Digital rectal examination

24. Pain control

25. Night sedation

26. Subcutaneous injection

27. Intramuscular injection

28. Intravenous injection

29. Venepuncture

30. Setting up and performing an IV cannulation

31. Setting up a transfusion and IV fluids

32. Mixing and injection drugs into an IV bag

33. Basic CPR

34. Airway management

35. ECG interpretation of AMI, unstable arrhythmias

36. Suturing

37. Removing sutures

38. Urinary catheter (male)

39. Urinary catheter (female)

40. Inserting a nasogastric tube

41. Arterial blood gases (sampling and interpretation)

42. Prepare sick certificate

43. Prepare worker’s compensation certificate

44. Prepare death certificate

45. Prepare cremation certificate

46. Obtain consent for procedures and investigation

Subscale Items

Clinical skills 1, 3–10, 12, 13, 16–21, 23

Procedural skills 14, 22, 26–30, 35–41

Operational management skills 2, 11, 15, 24, 25, 31–34

Administrative tasks 42–46

**Part 2: -** Please indicate the level at which you believe that medical school prepared you to:

1. Cope with stress caused by my work

2. Recognise my own clinical limitations

3. Discuss health risk behaviours with patients

4. Cope with my own emotions in distressing clinical situations

5. Discuss relevant preventative health strategies with patients

6. Take a drug and alcohol history with an initial consultation

7. Balance my work and professional life

8. Encourage patients to improve their health habits

9. Deal confidently with ‘difficult’ patients

10. Feel competent to tell a patient they have a terminal illness

11. Remain calm in difficult situations

12. Appreciate the importance of group dynamics when working within a team environment

13. Feel competent to counsel a distraught patient

14. Use opportunities to encourage patients to adopt healthier lifestyles

15. Be sensitive to the needs of nursing staff

16. Provide education to patients and families

17. Deal with dying patients

18. Approach confidently senior staff for help in interpreting investigations

19. Co-ordinate a comprehensive patient management plan with allied health professionals (e.g. physiotherapists)

20. Liaise with the social worker about my patients when necessary

Subscale Items

Interpersonal skills (Communication) 9, 10, 13, 17

Confidence (Reflective practice) 1, 2, 4, 7, 11, 18

Collaboration (Teamwork) 12, 15, 19, 20

Prevention (Social factors relevant to health) 3, 5, 6, 8, 14, 16

## Appendix 2: NSW prevocational progress review form end of term summative assessment (PRF-ETSA)

**Section 2:** To be completed by the term supervisor and the trainee

1.1. Demonstrates and applies knowledge of basic and clinical sciences

1.2. Obtains and presents history accurately

1.3. Performs appropriate clinical examinations

1.4. Ensures effective transition of patient handover

1.5. Acknowledges own limitations and seeks assistance when appropriate

1.6. Manages common problems and conditions

1.7. Recognises and assesses acutely ill patients and acts appropriately

1.8. Demonstrates ability to perform procedures

2.1. Demonstrates good communication with patients and family

2.2. Shows respect for patients and their decisions

2.3. Demonstrates appropriate written communication skills

2.4. Gives comprehensive case presentation

2.5. Appropriately completes medical records, including discharge summaries

2.6. Communicates with other medical staff and works effectively within the team

3.1. Demonstrates professional responsibility

3.2. Demonstrates ethical practice

3.3. Demonstrates good time management

3.4. Demonstrates commitment to continuous learning and development

4.1. How would you rate overall performance in this term?

Graduate Capabilities Subscale Items

Teamwork (Collaboration) 2.6

Reflective Practitioner (Confidence) 1.5, 3.1

Effective Communication (Interpersonal skills) 1.4, 2.1, 2.3, 2.4, 2.5

Ethically responsible actions 2.2, 3.1, 3.2

Self-directed learning 3.3, 3.4

Patient assessment and management 1.2, 1.3, 1.6, 1.7, 1.8

Basic and Clinical Sciences 1.1

## Pre-publication history

The pre-publication history for this paper can be accessed here:

http://www.biomedcentral.com/1472-6920/12/23/prepub
